# Development of a bio-inkjet printed LAMP test kit for detecting human African trypanosomiasis

**DOI:** 10.1371/journal.pntd.0008753

**Published:** 2020-10-22

**Authors:** Kyoko Hayashida, Peter Nambala, Nick Van Reet, Philippe Büscher, Naoko Kawai, Mable Mwale Mutengo, Janelisa Musaya, Boniface Namangala, Chihiro Sugimoto, Junya Yamagishi

**Affiliations:** 1 Division of Collaboration and Education, Research Center for Zoonosis Control (CZC), Hokkaido University, Sapporo, Japan; 2 International Collaboration Unit, Research Center for Zoonosis Control, Hokkaido University, Sapporo, Japan; 3 Department of Pathology, College of Medicine, University of Malawi, Blantyre, Malawi; 4 Department of Biomedical Sciences, Institute of Tropical Medicine, Antwerpen, Belgium; 5 Institute of Basic and Biomedical Sciences, Levy Mwanawasa Medical University, Lusaka, Zambia; 6 Department of Paraclinical Studies, School of Veterinary Medicine, University of Zambia, Lusaka, Zambia; University of Glasgow, UNITED KINGDOM

## Abstract

Human African trypanosomiasis (HAT) is one of the neglected tropical diseases in sub-Saharan Africa. Early diagnosis and treatment prior to disease progression are crucial for the survival of HAT patients. We had previously established a loop-mediated isothermal amplification (LAMP) method for HAT diagnosis in which the reagents were dried for field-use purposes. In this study, we used a semi-automated process to produce the test tubes using a bio-inkjet printer to achieve an accurate production. The performance of the inkjet printer-produced dried LAMP test (CZC-LAMP) was found to be stable after storage for up to 180 days at 30 °C. The diagnostic accuracy of CZC-LAMP HAT was evaluated using DNA samples that were extracted from 116 *Trypanosoma brucei gambiense* patients and 66 *T*. *b*. *rhodesiense* patients. The sensitivity was 72% for *T*. *b*. *gambiense* (95%CI: 63%–80%) and 80% for *T*. *b*. *rhodesiense* (95%CI: 69%–89%). The specificity determined using DNA from 116 endemic control DNA samples was 95% (95%CI: 89%–98%). The performance of the CZC-LAMP HAT and CZC-LAMP rHAT were also evaluated using 14 crude blood lysate samples obtained from *T*. *b*. *rhodesiense* patients and endemic control samples collected from Rumphi District in Malawi. The sensitivity and specificity were both 100% (95%CI: 77%–100%). As the developed CZC-LAMP test does not require a cold chain or a sophisticated laboratory, it holds promise for use as a routine simple molecular tool for point-of-care HAT diagnosis in endemic areas.

## Author summary

Nucleic acid amplification methods allow for a sensitive detection and specific identification of a number of pathogens, including *Trypanosoma* parasites that cause human African trypanosomiasis (HAT). However, conventional molecular tools such as the polymerase chain reaction (PCR) are highly resource-demanding and time-consuming and are seldom used in HAT-endemic countries. The loop-mediated isothermal amplification method (LAMP) provides a promising alternative to the conventional PCR, as this technique can amplify nucleic acids under isothermal conditions using minimal resources within one hour. In this study, we established a protocol to produce a dried-format LAMP kit for the diagnosis of HAT, that used a bio-ink-jet printer machine. The produced test kit (CZC-LAMP HAT and CZC-LAMP rHAT) was determined to be stable for up to 180 days at room temperature. The test provided a good performance for the detection of the trypanosome parasites using both DNA and crude blood samples, and the test results were comparable to those obtained from PCR. The findings from this study suggest that the dried LAMP test can be useful for the diagnosis of HAT, particularly in areas where laboratory resources are limited.

## Introduction

Human African trypanosomiasis (HAT), also known as sleeping sickness, is a neglected tropical disease that has previously caused devastating pandemics in Sub-Saharan countries [1]. As a result of extensive efforts to control the transmission of HAT by reducing the vector population, the tsetse fly (*Glossina* spp.), in combination with increased screening and treatment of parasite-infected people, HAT cases have dramatically declined. The HAT elimination goal, which aims to eliminate the disease as a public health problem by 2020, and to achieve zero cases of *Trypanosoma brucei gambiense* by 2030, was established by the World Health Organization (WHO) in 2012. This roadmap has progressed considerably, as less than 1,000 cases of HAT have been reported in 2018 [2]. This remarkable progress in HAT reduction can largely be attributed to the control of one of the two forms of HAT, gambiense HAT (gHAT) caused by *T*. *b*. *gambiense* in West and Central Africa, which accounts for 98% of the HAT cases [2]. gHAT typically presents with chronic symptoms that can persist for several years. The epidemiology of gHAT is maintained predominantly as an anthroponotic transmission from humans to humans, although the potential role of animals in the transmission cycle cannot be completely ruled out [3]. The use of mobile teams for active detection of gHAT by serological screening methods followed by microscopic examination, has resulted in tremendous success in the control of the disease. The card-agglutination test for trypanosomiasis (CATT) is used as the main method for mass-screening of populations at risk [4]. As the number of cases declines, immunochromatography-based rapid diagnostic tests (RDTs) have subsequently become available for point-of-care (POC) diagnosis. SD Bioline HAT (Abbott Diagnostics, South Korea) [5] and HAT Sero-K-SeT (Coris Bioconcept, Belgium) [6, 7] are RDTs detecting antibodies against native *Trypanosoma* VSG antigen LiTat1.3 and LiTat1.5. More recently, recombinant protein-based SD Bioline HAT 2.0 was also developed (Abbott Diagnostics and FIND) [8]. These RDTs showed good sensitivity ranging from 59.0% to 99.6% [6–9].

Another form of HAT, rhodesiense HAT (rHAT) cases caused by *Trypanosoma brucei rhodesiense* in Eastern and Southern Africa remains relatively low, fluctuating below 200 cases per year [2]. As CATT and available RDTs detect antibodies specific for the LiTat 1.3 and LiTat 1.5 antigen that is not generally expressed in *T*. *b*. *rhodesiense* [10], the detection of rHAT is not accurate using these RDTs, and instead relies largely upon microscopic examinations. Compared to gHAT, the disease progression of rHAT is typically rapid and involves parasite invasion of the central nervous system soon after the infection, ultimately resulting in the fatal second stage of the disease. Although less toxic drugs such as eflornithine and nifurtimox combination therapy (NECT) for the second stage of gHAT [11, 12] or more recently fexinidazole for the treatment of first and non-severe second-stage gHAT [13] have been developed, these drugs have not been approved for the treatment of rHAT. For the second-stage treatment of rHAT, melarsoprol is used, and this drug often causes severe adverse effects [14]. Thus, the early detection and prompt treatment of patients using less toxic drugs such as pentamidine or suramin during the first stage of the infection are critical for the effective treatment of HAT. rHAT is known as a typical zoonotic disease that is transmitted from domesticated and wild animals to humans. Due to its zoonotic nature, control of rHAT is difficult and hence elimination is more complicated as compared to gHAT [2]. Most rHAT cases have been reported in tourists who visited National Parks or local people living at the human-wildlife interface. Currently, four million people living within a 90,000 km2 area remain at risk of contracting rHAT [15, 2].

For the diagnosis of HAT, nucleic acid amplification methods detecting parasite DNA are another option for sensitive and specific detection of both forms of HAT [16]. Loop-mediated isothermal amplification (LAMP) is the isothermal amplification method that uses a strand-displacing *Bst*-polymerase with four to six primers. Minute amounts of the parasite’s nucleic acids can be rapidly detected using minimal resources. Dried-format ready-to-use LAMP diagnostics for HAT have been developed commercially (Loopamp *Trypanosoma brucei* assay; Eiken Chemical Co LTD, Japan) [17,18], based on the published primer sets targeting the repetitive insertion mobile elements (RIME) [19]. Using RIME-LAMP, both gHAT and rHAT can be diagnosed. As the Loopamp kit relies on a chelating agent for product visualization, the DNA extraction step is mandatory prior to the LAMP reaction, particularly when EDTA is used as an anti-coagulant. Since animals and tsetse-flies often harbor non-human infective *T*. *b*. *brucei* which cannot be distinguished by the RIME-base detection methods, a rHAT-specific LAMP was also established that can detect *T*. *b*. *rhodesiense* based on a unique serum resistance-associated gene (SRA) [20]. However this test is yet to be commercialized, and its dried-format reagent has not been developed.

Previously, we established an in-house protocol to dry the LAMP reagents using trehalose and glycerol as preservatives [21]. In the presence of trehalose, the liquid solution is altered to a sugar-glass or to an amorphous phase in which the molecules can maintain stability [22]. The enzyme stability in trehalose sugar-glass is further increased by adding glycerol [23]. In a previous study, we optimized the concentration of trehalose and glycerol in a manner that allowed the enzyme activity to remain intact, and the sugar-glass was maintained at an ambient temperature [21]. The established protocol was also used to develop the LAMP tests targeting other tropical diseases such as malaria [24], chikungunya [25], and camel trypanosomiasis caused by *Trypanosoma evansi* [26]. The DNA intercalator GelGreen was used to visualize the LAMP product to allow crude biomedical materials such as detergent-lysed blood to be used for the reaction. In contrast to the conventional LAMP test that requires a cold chain and handling by skilled personnel, the established ready-to-use dried-format LAMP possesses advantages for use in the field setting. In these LAMP kits, the enzyme was placed on a tube lid and air-dried under clean air or dried using an ultra-low dew point air drier [25]. However, the success of dry-LAMP production was highly dependent upon the efficacy of the drying procedure used to maintain the enzyme activity. Since the reagents were dispensed manually by pipette, quality control of the reagents was also the issue left unsolved.

In this study, we adapted a bio-inkjet printer machine to produce a standardized dried LAMP test kit for the detection of gHAT and rHAT. Reagents were “printed” onto a reaction tube lid, and these semi-automatically produced kits were tested for stability over 180 days. The diagnostic performance and accuracy of the produced test tubes was evaluated using purified DNA samples from confirmed *T*. *b*. *gambiense*, *T*. *b*. *rhodesiense* HAT cases, and endemic controls. Additionally, crude blood samples from recent *T*. *b*. *rhodesiense* infection cases in Malawi were also evaluated, and the results of our study confirmed the suitability of our test for HAT diagnosis and epidemiological surveys.

## Methods

### CZC-LAMP preparation

A bio inkjet printer (LaboJet-500Bio; Microjet, Nagano, Japan) was used to spot *Bst*-polymerase with trehalose on a flat tube cap strip (#TCS0803; Bio-Rad, Japan). Four strips consisting of eight caps for each were arrayed on the stage with a plastic holder. The enzyme solution consisted of 0.086 μL of a high-concentration *Bst*-polymerase (120 U/μL; New England Biolabs Inc., Ipswich, MA, USA), 0.369 μL of trehalose (2 mol/L = 2 M in water; FUJIFILM Wako Pure Chemical, Osaka, Japan), and 0.228 μL of nuclease-free water per one tube, were prepared. The inkjet printer head was filled with a total of 0.6825 μL of an enzyme solution per test, and droplets were printed onto the tube caps. The tube caps were then removed and stored with the bottom tubes as described below. The dNTP/CFI/primer mixture consisted of 1.4 μL of dNTPs (25 mM; Nippongene, Japan), 1 μL of CFI, 0.4 μL of the FIP and BIP primers (100 mM), 0.01 μL of the F3 and B3 primers (100 mM), 0.2 μL of the LF and LB primers (100 mM), and 0.7 μL of trehalose (2 M in water) in each test tube. The CFI consisted of 3 mM hydroxyl-naphtol blue (MP Biomedicals, Aurora, OH, USA) and 0.35% v/v GelGreen (10,000 × Sol, Biotium, Hayward, CA, USA). RIME primer sequences and SRA primer sequences have been described previously [21], which were slightly modified from the primer sets reported by Njiru et al., [19] to achieve an improved sensitivity for use on the southern African type rHAT. The dNTP/CFI/primer mixture was aliquoted into 0.2-mL PCR strips (#TLS0801; Bio-RAD, Japan) using an electronic pipette (E1-ClipTip; Thermo Fisher Scientific) and then placed in the glove box connected to an ultra-low dew point air dryer (QD20-50; IAC Co., Kawasaki, Japan) for 12 h. The enzyme-spotted tube lid and the dNTP/CFI/primer mixture containing tube bottom were stored with 1 g of the molecular sieves 3A (FUJIFILM Wako Pure Chemical) in an aluminum bag. In this manuscript, six batches of test tubes were used. Batch #A for stability test, batch #B for the test of DNA samples, batch #C for the test of blood samples in Malawi, and batches #D/#E/#F for the batch variability tests. For the stability tests, the bags containing batch #A LAMP tests were stored in a humid chamber (IG4-1; Yamato Scientific, Tokyo, Japan) set to 50% RH and 30 °C for up to 180 days. For batch #B, LAMP test using DNA samples was conducted ten days after storage at room temperature. For batch #C, LAMP test using blood sample was conducted after five months storage at room temperature. For batches #D, #E, and #F for the inter-batch variability evaluation test, LAMP tests were conducted after 15–17 days storages at room temperature. For batch #B and batch #C, the performance of HAT- and rHAT- CZC-LAMPs were confirmed after the production and before tests using 10 (for RIME) and 100 (for SRA) parasite eq., and both batches detected these limits of detection. Sensitivity, specificity, agreement values, and statistical significance were determined using epiR and irr packages in R software (https://CRAN.R-project.org).

### Laboratory confirmation of the sensitivity, specificity, and inter-batch variability using real-time PCR machine

Dried CZC-LAMP reagents were reconstituted with 23 μL of the reaction buffer (20 mM Tris-HCl, pH 10.0, 50 mM KCl, 6 mM MgSO_4_, and 10 mM [NH_4_]_2_SO_4_, 0.1% Trion X-100) by inverting the tubes and mixing thoroughly. Two microliters of the DNA templates were added to each reaction. Amplification was monitored using a real-time PCR machine (CFX-96; Bio-Rad) using the FAM channel that detected the fluorescence of GelGreen in the CZC-LAMPs. One cycle of amplification was set as one minute, and thus, the threshold cycle (Ct) values at the were considered to be indicative of the reaction speed (min) of the LAMP reaction. After 100 cycles of amplification at 63 °C, melting curve analysis was performed. A parasite isolate from the Zambian rHAT patient (*T*. *b*. *rhodesiense* UTH2012) [27], was used as the laboratory confirmation template. The parasite (0.5 x 10^6^) were lysed in 1 mL of 0.1% Triton X-100 in distilled water, and this was calculated as 1000 parasites equivalents per reaction (eq./reaction).

### DNA samples from gHAT and rHAT patients and laboratory tests

The DNA samples from gHAT and rHAT patient samples were stored at the Institute of Tropical Medicine (ITM), Antwerp, Belgium. DNA was extracted from the stored blood samples. *T*. *b*. *gambiense* specimens (n = 116) and the same number of healthy endemic control samples were collected from the Democratic Republic of the Congo in 2010. *T*. *b*. *gambiense* samples were microscopically parasite-confirmed in the blood, lymph, or cerebrospinal fluid (CSF) samples using parasite concentration methods as described by Mitashi et al., [18]. *T*. *b*. *rhodesiense* specimens (n = 77) were obtained with permission from the WHO HAT specimen bank, that is the specimen bank used for research to develop HAT diagnostics. The samples originated from Uganda, Tanzania, and Malawi and were collected from 2008 to 2012 [28]. The CZC-LAMP HAT was used to assess these DNA samples from gHAT and rHAT patients. Reaction times and specificity were monitored using a real-time PCR machine (QuantStudio5; Thermo Fisher Scientific), and 60 min was set as the endpoint for the reaction. Polymerase chain reaction (PCR) for trypanosome detection targeting the 18SrRNA gene (M18S-II-Tb-PCR) was performed in accordance with methods described in a previous study [29].

### Blood samples from rHAT patients in Malawi and laboratory tests

Blood samples from rHAT patients (n = 14), and the same number of the control blood samples were obtained from villages in Rumphi District, Malawi. These rHAT cases were identified through active and passive surveys between May 2016 and December 2016, under the project of TrypanoGEN at the H3Africa Consortium [30, 31]. For the diagnosis of rHAT, blood was centrifuged in a capillary tube and the buffy-coat was examined under a microscope [32]. If the trypanosomes were observed in the blood, CSF was collected from the patient and examined for presence of parasites (stage 2 infection). The blood was stored at −20 °C freezer at Rumphi District Hospital. Later the samples were transferred to the College of Medicine, Malawi, for the CZC-LAMP HAT/rHAT tests, DNA extraction, ITS1-PCR, and SRA-PCR tests. DNA was extracted from 200 μL of blood using a DNA Isolation kit for mammalian blood (Roche Diagnostics GmbH, Mannheim, Germany). ITS1-PCR was performed to detect *T*. *b*. *gambiense* [33] and *T*. *b*. *rhodesiense*, while SRA-PCR was used for the detection of *T*. *b*. *rhodesiense* [34]. Each 20 μL reaction mixture contained the Ampdirect Buffer (Shimadzu, Kyoto, Japan), 10 μM of the forward and reverse primers [34, 35], 0.5 U of the BIOTAQ HS DNA polymerase (Bioline, London, UK), and 2 μL of the template DNA. Amplification conditions were as follows: 95°C for 10 min, 40 cycles of denaturation at 95°C for 30 s, annealing at 60°C (SRA-PCR) or 57°C (ITS1-PCR) for 1 min, denaturation at 72°C for 2 min, and final extension at 72°C for 5 min.

CZC-LAMP HAT and CZC-LAMP rHAT tests were performed using 2 μL of the extracted DNA or 10 μL of the lysed blood template. The lysed blood was prepared by diluting ten times with 0.1% Triton X-100 in water. The LAMP test tubes were incubated in a portable incubator (BSR-miniT100H, Bio Medical Science, Tokyo, Japan) at 63°C for 30 min for CZC-LAMP rHAT and 60 min for CZC-LAMP HAT, respectively. After the LAMP reactions, positive fluorescence was observed using a portable LED illuminator under a 500 nm light [21, 35].

### Ethical statement

DNA samples from gHAT and rHAT that were stored at ITM, were obtained from a previous study published by Mitashi et al. [18]. Written informed consent from the participants was obtained, and all samples were anonymized. The use of the stored samples for this HAT diagnostic study was approved by the ethical committees of the University Hospital in Antwerp (ITG09415684, B30020108363) and the Ministry of Health of the Democratic Republic of Congo (M-D/226/2010 and M-D/179/2010).

The study performed at Rumphi received ethical clearance from the Malawi National Health Sciences Research Committee (NHSRC 15/4/1399, approved on 2015 April 17). Written informed consent was obtained from each participant in the presence of an independent witness.

## Results

### Production of CZC-LAMP HAT and CZC-LAMP rHAT

**Fig 1:**
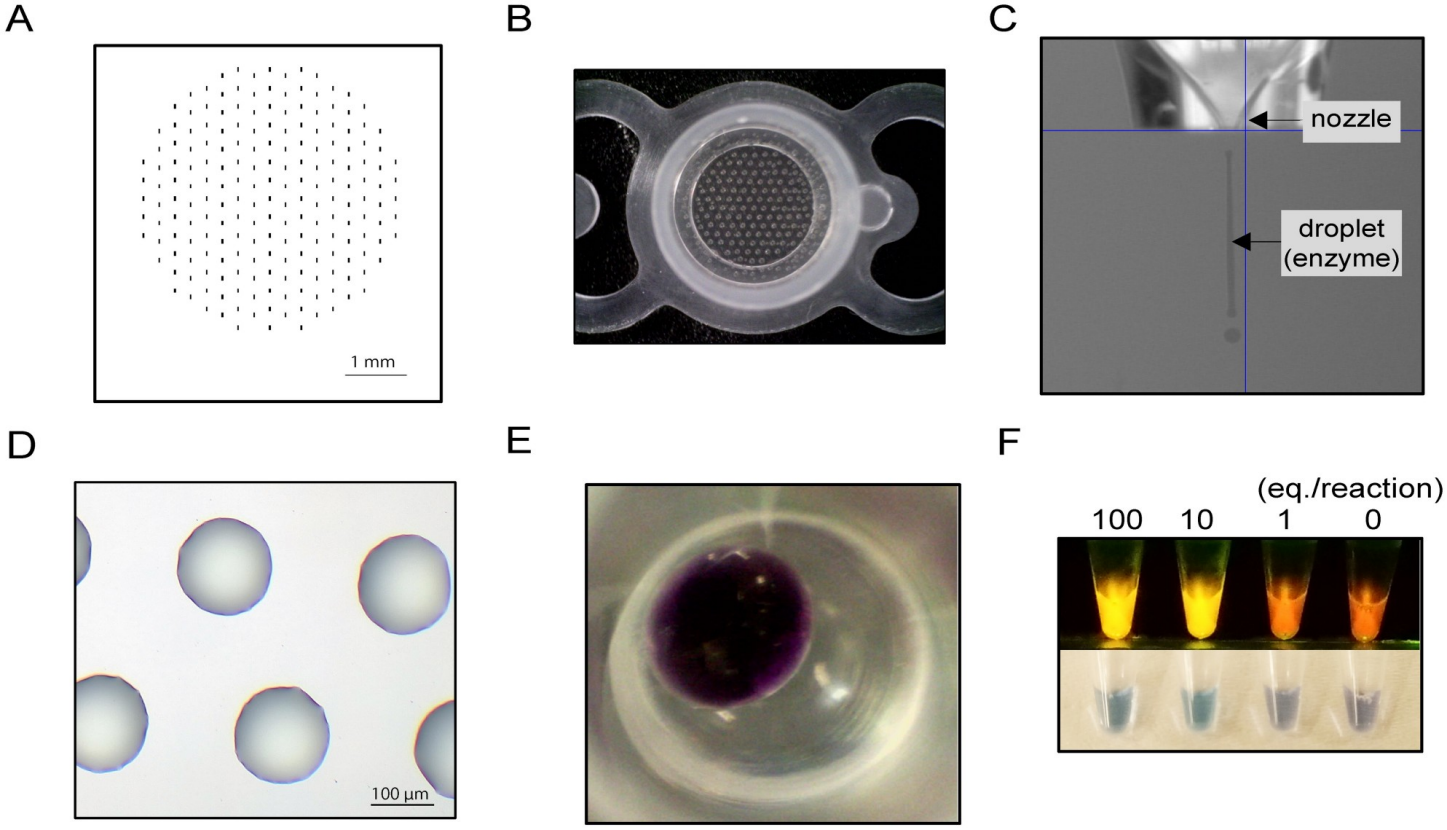
Dried format CZC-LAMP was produced by using a bio-inkjet printer. A) The bitmap file template used for bio-inkjet printing on the tube lid. B) LAMP test tube lid after printing of the enzyme. C) Image of the inkjet printer head while ejecting reagents. D) Microscopic image of LAMP test tube lid showing the regular shape of the sugar-glass array. E) Appearance of the tube bottom. The primers, dNTPs, and detection dye CFI were vitrified with trehalose. F) Representative image of the positive and negative results of CZC-LAMP HAT observed under LED light (upper) and by the naked eye (bottom). CZC-LAMP HAT reactions were incubated for 60 min at 63 °C with DNA at the indicated equivalent parasite concentration (eq./reaction).

Dried-format LAMP test tubes were produced, that contained *Bst*-polymerase on the lid, and the remaining reagents (primers, detection dye, and dNTPs) on the tube bottom. To enable large-scale and accurate production, a bio-inkjet printer was utilized for the test tube production, which is referred to as CZC-LAMP. *Bst*-polymerase in combination with trehalose was “printed” on the tube lid at 213 dots per tube (Fig 1A and 1B). This bio-inkjet printer uses piezoelectric strategy to eject the liquid, where the liquid is ejected by a voltage-induced deformation. Efficient ejection from the printer nozzle tip was achieved by diluting the enzyme and trehalose in water, which serves to reduce the viscosity of the solution. From the piezoelectric head nozzle, the droplets with volume of 3.2 nL were ejected and monitored throughout the production by a camera equipped within the device (Fig 1C). After printing, the printed solution was rapidly dried, and the formation of a non-crystalline sugar-glass was observed soon after the production (Fig 1D). Primers, detection dye colori-fluorometric indicator (CFI) [21], and dNTPs with trehalose were placed at the bottom of the tubes, and the tubes were dried using an ultra-low dew point air drier [25] (Fig 1E). Two sets of the primers were used, with one detecting both rHAT and gHAT using the RIME primer set (CZC-LAMP HAT) and the other detecting rHAT specifically using the SRA primer set (CZC-LAMP rHAT). The visual appearance of CZC-LAMP after the reaction is shown in Fig 1F, where positive samples produced a yellowish fluorescence and negative samples exhibited an orange color under 500-nm LED light (upper panel). Upon visual inspection, the color of the positive tubes turned to sky-blue from purple due to the presence of hydroxy naphthol blue in the detection dye CFI that allows for the detection of magnesium consumption and pH change after LAMP reactions [36]. For the stability and validation tests using DNA, the change in fluorescence was determined using a real-time PCR machine.

### Laboratory test for the CZC-LAMP stability

**Fig 2:**
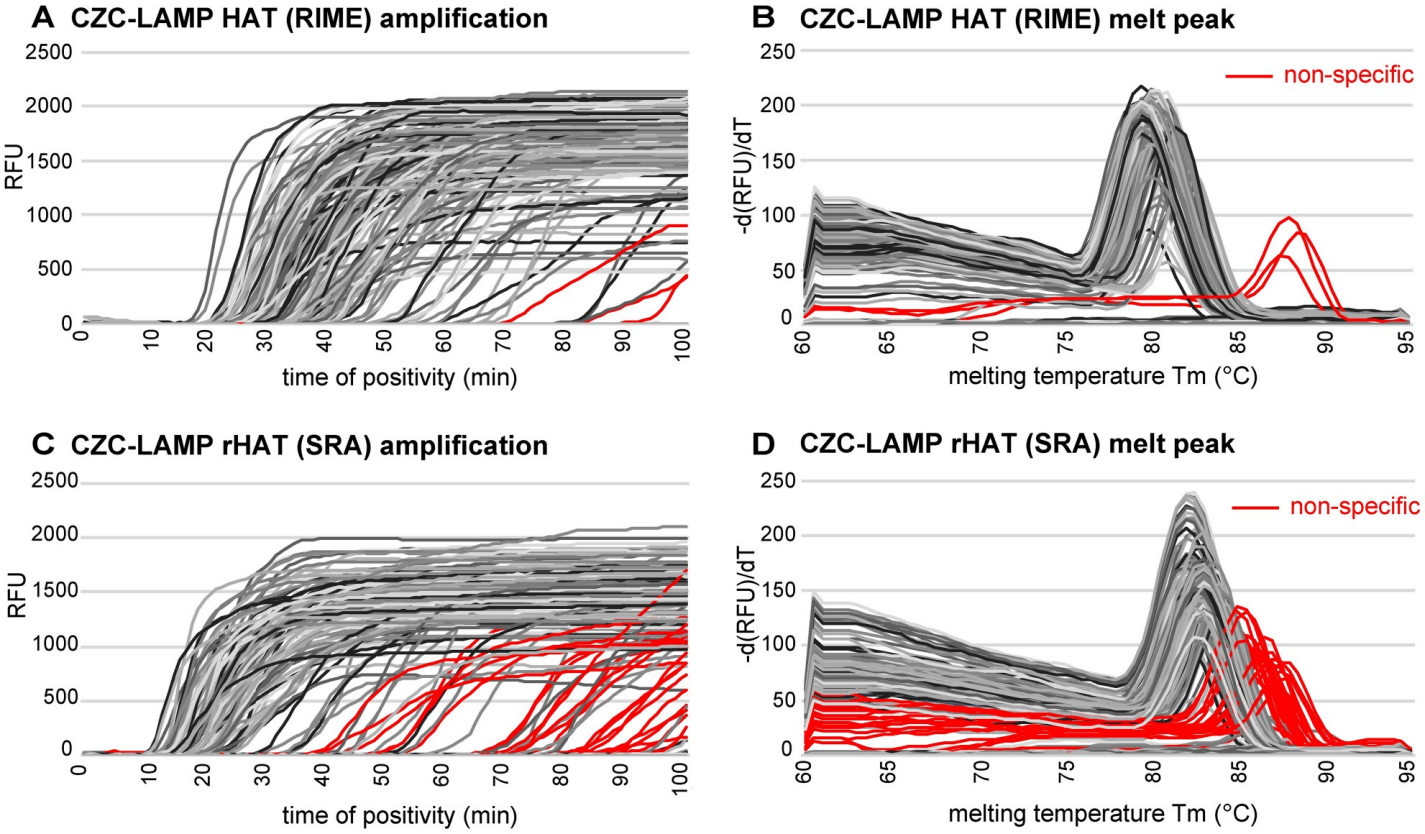
Amplification and melting curves of CZC-LAMP HAT (RIME) and CZC-LAMP rHAT (SRA). A) Real-time amplification fluorescence signal of 214 tests of CZC-LAMP HAT. B) Melting curves of CZC-LAMP HAT products. The curves showed melting temperature (Tm) of 80.0 ± 0.61 °C (average ± 2SD, calculated with 100, 10 eq. test results). C) Real-time amplification fluorescence signal of 214 tests of CZC-LAMP rHAT. D) Melting curves of CZC-LAMP rHAT products. The average melting temperature (Tm) was 82.9 ± 0.68 °C (average ± 2SD, calculated with 1000, 100 eq. test results). Red colors show out of range results, suggesting non-specific amplification.

CZC-LAMP HAT and CZC-LAMP rHAT were stored for 7 days, 30 days, 90 days, and 180 days at 30°C. Half of the test tubes were stored in closed bags containing desiccants to facilitate dry storage conditions (30°C-dry). The other half of the test tubes were stored without desiccants, and the tubes were exposed to 50% relative humidity (RH) conditions during storage (30°C, 50%RH). The performance of CZC-LAMPs after storage under each condition was evaluated using detergent-lysed *T*. *b*. *rhodesiense* as a template, and analytical sensitivity was determined. Amplification curves and melting curves of 214 tests of CZC-LAMP HAT (RIME primer) and CZC-LAMP rHAT (SRA primer) were obtained by using real-time PCR machine (Fig 2). For each primer set, one major melting temperature (Tm value) was obtained. However, some products amplified from a lower concentration of template or no target control (NTC) showed higher temperature peaks (Fig 2B and 2D). These reactions were most probably due to non-specific amplification events involving primer dimers [37]. The Tm values within average ±2 SD were treated as specific amplification. The non-specific amplifications (false positives) were shown in red colors (Fig 2).

**Fig 3:**
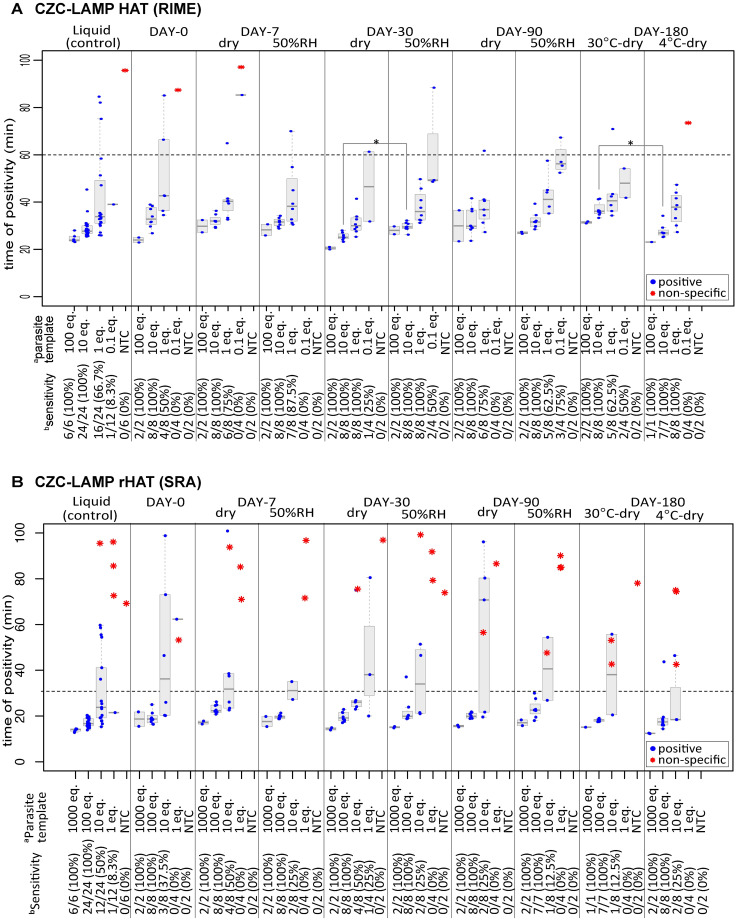
Analytical sensitivities of CZC-LAMPs after storage. (A) CZC-LAMP HAT (RIME primer set) and (B) CZC-LAMP rHAT (SRA primer set) test tubes were stored in dried or 50% relative humidity (RH) environments at 30 °C for 0–90 days, or at 4 °C or 30 °C under dry condition for 180 days. The time of positivity and specificity according to melting temperature (Tm value) were analyzed using real-time PCR machine. The genuine positive reaction time points according to the Tm values are shown as blue plots, while non-specific amplifications are plotted as red asterisk. The dashed line presents the threshold of the reaction time to calculate sensitivity. Conventional liquid LAMP (liquid) was used as the reaction control. ^a^ Indicated equivalent numbers of *Trypanosoma brucei rhodesiense* lysate per reaction (eq.) were used as templates. ^b^The positivity was judged at the 60-min (A; CZC-LAMP HAT) or 30-min (B; CZC-LAMP rHAT) incubation time threshold. Statistical significance was assessed using Mann-Whitney U test (*p<0.001).

The LAMP reaction sensitivities presented in Fig 3A and 3B were calculated at 60 min and 30 min reaction time points for CZC-LAMP HAT and CZC-LAMP rHAT, respectively. We used these reaction time thresholds based on the reaction speed range with templates while leaving enough time until non-specific amplification (red asterisk) was observed (Fig 3). LAMP using the RIME primer set in the liquid format as a reaction control, exhibited 100% sensitivity (24 of 24, 95% CI: 86%–100%) in 10 parasites-equivalents template lysates (10 eq./reaction). The detection limit of our produced CZC-LAMP HAT possessed the same sensitivity as that of the control liquid LAMP, exhibiting 100% sensitivity (8 of 8, 95% CI: 63%–100%) in 10 eq./reaction template during 180 days of storage (Fig 3A). Similarly, no decrease in the sensitivity of CZC-LAMP rHAT detecting SRA was observed, as sensitivity remained at 100% (8 of 8, 95%CI: 63%–100%) at 100 eq./reaction during 180 days of storage (Fig 3B). The analytical sensitivities of the endpoint analysis were also the same between the test tubes stored at 4 °C and 30 °C for 180 days. However, the reaction time was significantly faster in 4 °C stored test tubes compared to 30 °C stored tubes (Fig 3A, DAY-180, 10 eq.: median reaction times: 36 min in 30 °C vs 27 min in 4 °C, p<0.001). The sensitivity was also higher in 4 °C stored tubes compared to 30 °C stored tubes (Fig 3A, DAY-180, 1 eq.: 100% in 4 °C vs 62.5% in 30 °C; Fig 3B, DAY-180, 10 eq.: 25.0% in 4 °C vs 12.5% in 30 °C). Also, tubes stored in 50%RH condition showed delayed reaction time compared to dry condition (Fig 3A, DAY-30, 10 eq.: median reaction times: 30 min in dry vs 32 min in RH50%, p<0.001), and sensitivity was also slightly decreased (Fig 3A, DAY-90, 1 eq., 75% in dry vs 62.5% in 50%RH; Fig 3B, DAY-90, 10 eq.; 25% in dry vs 12.5% in 50%RH).

To assess inter-batch variability, three independent batches of CZC-LAMPs were tested (batches #D, #E #F). The determined sensitivities were comparable to that observed in the tubes used for the stability test (batch #A), detecting up to 10 and 100 parasite eq. in the 100% sensitivity (8/8), suggesting that the production procedures were reproducible (S1 Fig).

### Validation of diagnostic accuracy using DNA samples from the gHAT and gHAT patients

**Fig 4:**
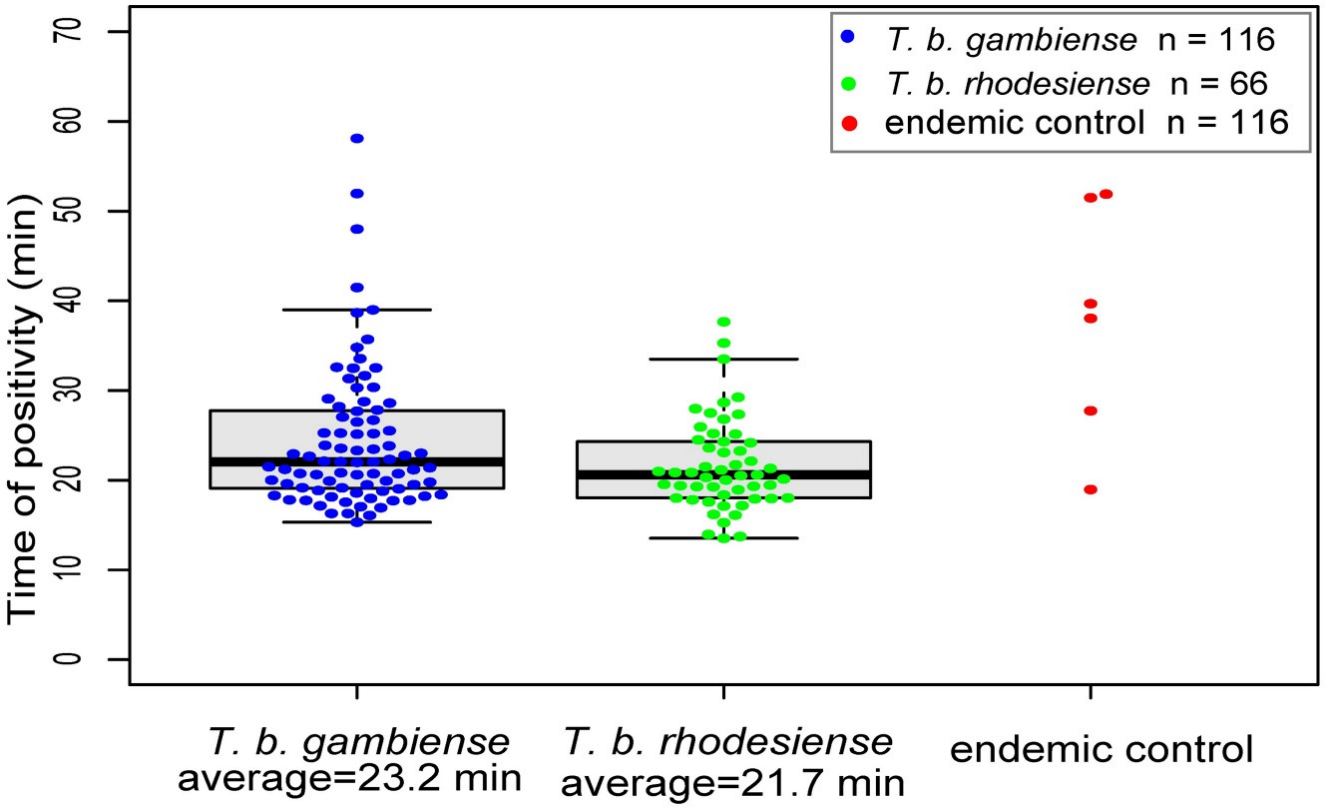
Time of positivity of CZC-LAMP HAT test in the HAT DNA samples. CZC-LAMP HAT was tested for 116 *T*. *b*. *gambiense* clinical samples, 66 *T*. *b*. *rhodesiense* samples, and 116 endemic control samples. The time of positivity was monitored by real-time PCR machine and is shown as blue, green, and red dots. A non-specific reaction was not observed at the 60-min time point.

To evaluate the diagnostic accuracy of the CZC-LAMP HAT assay to detect *T*. *b*. *rhodesiense* and *T*. *b*. *gambiense*, DNA samples obtained as described in a previous study [18] and from the WHO HAT specimen bank [28], were analyzed using the novel CZC-LAMP HAT test. Of the total 116 DNA extracts from *T*. *b*. *gambiense*-confirmed samples (gHAT DNA), 84 samples were positive, demonstrating a sensitivity of 72% (95%CI: 63%–80%) (Table 1). Of the 116 healthy endemic control samples that were collected at the same time as the *T*. *b*. *gambiense* samples in the same village, six yielded a positive reaction in HAT-CZC-LAMP, indicating a 95% specificity (95%CI: 89%–98%). For DNA from *T*. *b*. *rhodesisense*-confirmed samples (rHAT DNA), 53 of the 66 tested samples showed a positive reaction in CZC-LAMP HAT, indicating an 80% (95%CI: 69%–89%) sensitivity. The average reaction speed of all positive reactions in gHAT DNA and rHAT DNA were 23.2 min and 21.7 min, respectively (Fig 4). Although the LAMP reaction speed does not provide exact quantitative information, a higher amount of template was typically more rapidly amplified as shown in Fig 3. Therefore, faster reaction speed in rHAT DNA likely resulted from a higher number of parasites in the blood of rHAT patients.

**Table 1:** CZC-LAMP HAT test results using the DNA extracted from rHAT, gHAT, and endemic control samples, and its agreement with the PCR test.

Sample		LAMP positive	LAMP negative	
*T*. *b*. *gambiense* (gHAT) patient DNA (n = 116)^a^		84	32	Sensitivity: 72% (95%CI: 63%–80%)
Healthy endemic control (n = 116)		6	110	Specificity: 95% (95%CI: 89%–98%)
*T*. *b*. *rhodesiense*^a^ (rHAT) patient DNA (n = 66)	PCR^b^ positive	51	2	Sensitivity: 80% (95%CI: 69%–89%)
	PCR^b^ negative	2	11	Agreement with PCR: 94% (k = 0.81)

^a^ The parasitological detection in the original specimen was considered to be true-positive.

^b^ M18S-II-Tb-PCR results were used for comparison with the results of CZC-LAMP HAT test.

To compare the diagnostic sensitivity and specificity of our CZC-LAMP HAT with the other molecular detection method, M18S-II-Tb-PCR targeting the 18SrRNA of *Trypanosoma* spp. was performed [29] using 66 *T*. *b*. *rhodesiense* samples. A total of 51 out of the 53 LAMP positive samples were also positive by PCR, while 11 of the 13 LAMP-negative samples were negative by PCR, demonstrating a 94% agreement with an excellent kappa value of 0.81 between the CZC-LAMP HAT and M18S-II-Tb-PCR test (Table 1).

### Validation of diagnostic accuracy using blood samples from rHAT patients in Rumphi, Malawi

Samples from recent rHAT cases in Rumphi, Malawi (n = 14), and the same number of endemic control samples were tested using CZC-LAMP HAT and CZC-LAMP rHAT. In this field experiment, detergent-lysed blood samples were used as templates, and the results were compared to the result from the extracted DNA templates. In the detergent-lysed blood samples, the sensitivity and specificity were 100% (95%CI: 77%–100%) for both CZC-LAMP HAT and CZC-LAMP rHAT (Table 2). However, in the extracted DNA samples, 4 out of the 14 HAT patient samples failed to be detected by CZC-LAMP rHAT. Among these four false-negative samples, SRA-PCR also yielded three negative results (CZC-LAMP rHAT vs SRA-PCR; kappa value of 0.924), suggesting that these false-negative samples contained only a small amount of DNA that was below the detection limit of CZC-LAMP rHAT and SRA-PCR.

**Table 2:** CZC-LAMP HAT/rHAT test results using the blood-direct method using rHAT specimens from Rumphi, Malawi, and its comparison with results of the PCR tests.

test	template	Rumphi rHAT			Rumphi endemic control		
		positive	negative	sensitivity	positive	negative	specificity
CZC-LAMP HAT	DNA	14	0	100% (95%CI: 77%–100%)	0	14	100% (95%CI: 77%–100%)
	blood	14	0	100% (95%CI 77%–100%)	0	14	100% (95%CI: 77%–100%)
CZC-LAMP rHAT	DNA	10	4	71% (95%CI: 69%–100%)	0	14	100% (95%CI: 77%–100%)
	blood	14	0	100% (95%CI: 77%–100%)	0	14	100% (95%CI: 77%–100%)
ITS1-PCR	DNA	14	0	100% (95%CI: 77%–100%)	0	14	100% (95%CI: 77%–100%)
SRA-PCR	DNA	11	3	79% (95%CI: 49%–95%)	0	14	100% (95%CI: 77%–100%)

## Discussion

A rapid, sensitive, low-cost molecular diagnostic test is necessary to achieve and monitor the elimination status of HAT in endemic countries. Monitoring of the parasite-reservoir animals and the vector tsetse-flies for presence of human-infective trypanosomes at the subspecies level is also essential to assess the risks of HAT infection within the community, and this can only be achieved by the nucleic acid detection molecular methods. As the majority of the endemic foci of HAT are in remote areas, cold-chain-free and field-applicable molecular diagnostics are desired. In this study, we applied a bio-inkjet printer technology [38] to produce a dried-format LAMP test for the detection of HAT caused by *T*. *b*. *gambiense* and *T*. *b*. *rhodesiense*. The idea to spray microdroplets was based on the findings from previous studies [21] that revealed that enzyme activity could be stably maintained in the trehalose sugar-glass only when the water was rapidly removed from the enzyme solution spotted onto the PCR lid. The accurate liquid handling of the nanoliter-scale reagents using a conventional pipette presents another concern, as accurate handling is of utmost importance when designing reliable test systems. The bio-inkjet printer used in this study enabled precise liquid handling at the nanoliter-scale, and the small amount of the liquid assisted faster dry up of the enzyme to create sugar-glass on the plastic PCR tube lid. We separately prepared enzyme (lid) by bio-inkjet printer and primers (tube bottom) by automatic pipette, thus the production procedure was still not fully automated. Ideally, all reagents could be prepared using bio-inkjet printer, which will make the fabrication process simpler. However, when the top and bottom reagents, which contain *Bst*-polymerase, dNTPs, CFI, primers, and trehalose were combined as one mixture, apparently delayed reaction time was observed. The probable explanation is that the enzyme will be exposed to high concentration of other reagents during drying procedure which may negatively affect enzyme activity. In addition, making a glassy amorphous phase for enzyme vitrification by drying procedure seems to be highly dependent upon the efficacy or speed of dehydration as observed in our experience and other reports [39, 40], thus higher volume of the reagents may simply delayed the speed of dehydration and may form crystalline trehalose which deteriorate the enzyme activity. The accuracies of inkjet printer and automatic pipette are considered to be more accurate because the procedures are independent of user experience [41, 42]. Indeed, three independent batches of CZC-LAMPs were tested for their sensitivities, resulting in similar performances in terms of reaction speed and sensitivity (S1 Fig). Observed variability between samples, batches, and the experiments may also be attributed to the technical differences during preparation and applying a template DNA by manual pipetting.

Our CZC-LAMP specific for gHAT and rHAT that are produced in this modified process were confirmed to be stable after storage at 180 days at 30°C. However, slightly decreased sensitivity and increased detection time were observed in the test tubes stored at 30°C or 50%RH compared to tubes stored at dry 4°C condition. These stability test results suggested that the test kits can be stored at 30 °C and 50%RH environments with acceptable performance, but cold and dry condition is more recommended for the long-term storage.

These cold-chain-free format LAMP tests provide a promising future for enhanced HAT diagnosis, as the cold-chain has presented the biggest bottleneck for the implementation of molecular diagnosis in the rural areas. The most reliable gold-standard diagnostic method to test for HAT is still a conventional parasitological confirmation test that uses microscopy combined with concentration of parasites by capillary tube centrifugation [32] or a mini anion exchange-column mAECT designed for a field use [43]. However, these procedures are time-consuming and labor-intensive, particularly when a large number of individuals are to be tested. The use of microscopy in rural health facilities for HAT diagnosis also presents several challenges such as the need for skilled personnel, difficulties in detecting low parasite density samples, and parasite morphology deterioration after storage at room temperature. Additionally, the expanded use of the malaria rapid diagnostic tests reduces the routine use of microscopy for disease diagnosis [44], and may decrease the chance of HAT diagnosis. For screening of gHAT in the field, serological tests such as CATT or RDT devices exist, but unfortunately, neither of these methods are applicable to rHAT, and serology alone cannot be used to diagnose an active infection [45]. Moreover, in both gHAT and rHAT, there is increasing evidence that healthy asymptomatic carriers exist [3, 45–47]. These latent infections are characterized as asymptomatic and with undetectable low parasite numbers in the peripheral blood. The latently infected people may function as reservoir, and hence may hamper the achievement of the elimination goals. Serologically negative and parasite DNA positive individuals have also been reported [47, 48]. Our observed six CZC-LAMP HAT-positives among the healthy endemic controls from the Democratic Republic of the Congo, might have been cases with a latent infection of *T*. *b*. *gambiense*. Although the epidemiological role of latently infected people is still unclear, the application of CZC-LAMPs as sensitive and affordable RDTs, in combination with existing antibody detecting RDTs have merit for the detection of these cases.

The analytical sensitivity was determined to be 100% in 100 parasite-equivalents template for CZC-LAMP HAT and 1,000 parasite-equivalent for CZC-LAMP rHAT per reaction tube. As we used one μL equivalent of blood for the reaction in the blood-lysis method, these observed detection limits were equivalent to 1 × 10^5^ parasites/mL and 1 × 10^6^ parasites/mL within the original blood for HAT and rHAT. This sensitivity of CZC-LAMPs was lower than that of the conventional counting chamber method, which typically detects up to 1 × 10^4^ parasites/mL [10]. In lower number of parasite templates, the detection of our LAMP systems became stochastic, and it detected up to 0.1 parasite-equivalent (1 × 10^2^ parasites/mL) and 1 parasite-equivalent (1 × 10^3^ parasites/mL) in CZC-LAMP HAT and CZC-LAMP rHAT, respectively. The determined sensitivities of CZC-LAMP HAT for *T*. *b*. *gambiense* and *T*. *b*. *rhodesiense* validated by the WHO HAT-Specimen Bank samples, were 72% (95%CI: 63%-80%) and 80% (95%CI:69%-89%), respectively, and the specificity calculated using healthy endemic controls was 95% (95%CI:89%-98%). The observed sensitivity and specificity were lower than the reported performance of the M18S-II-Tb-PCR test, where the sensitivity and specificity were 88.4% and 99.2%, respectively [29]. Nevertheless, the agreement between M18S-II-Tb-PCR and CZC-LAMP HAT was high, exhibiting 94.0% agreement with a kappa value of 0.81. The false negative CZC-LAMP HAT test results were most likely the result of low DNA concentration of parasites that were below the detection limit in both the PCR and LAMP methods. Low concentration of parasite DNA could be due to low parasitemia, sample deterioration, or a failure in DNA extraction. Although comparison of accuracy between diagnostic tests is difficult without simultaneous testing on the same samples, both analytical and retrospective patient DNA analysis showed lower sensitivity than parasitological examination. However, considering that CZC-LAMPs is detecting parasite DNA and can identify the *T*. *b*. *rhodesiense* subspecies, it may be useful for the confirmation of rHAT infection for which field-applicable serological tests are not available. The easier workflow and low-cost features of CZC-LAMPs are more suitable for field use than PCR. In the direct blood method where blood is lysed by detergents [21, 24, 25], DNA extraction is not required. The initial cost to introduce the LAMP test is minimal, as the equipment required for CZC-LAMPs for field usage is only an incubator or a water bath to maintain the reaction temperature at 63°C and an LED illuminator. The reagent cost to make CZC-LAMP was calculated as 0.86 US$ per test tube. This price is similar to other available RDTs that only provide serological suspicion of infection (SD Bioline HAT, 0.60$; CATT, 0.74$; HAT sero-K-SeT, 1.97$) [49]. Long shelf life of CZC-LAMP (over 6 months) at ambient temperature is also suitable for the use in clinics in remote areas. The rapid identification of parasites within 60 min at the subspecies level in blood or CSF samples will aid rapid HAT diagnosis and disease staging, and these will guide proper treatment decisions.

We also tested blood samples obtained from rHAT patients in Malawi. In the blood-lysis method, which uses the crude blood samples directly, all fourteen rHAT samples were positive in CZC-LAMP HAT and CZC-LAMP rHAT tests. The specificity determined by the fourteen endemic control was also 100%. When DNA was extracted from the same blood samples and tested for CZC-LAMPs, four samples were negative in CZC-LAMP rHAT test. Theoretically, DNA quality and quantity should be increased during extraction; however, possible technical errors during extraction cannot be ruled out. We suspect that the obtained false negatives in the four DNA samples were due to technical artifacts. This explanation could also be applied to the observed low sensitivity our test using DNA extracted from stored rHAT and gHAT patients. As the tested sample number of patient blood in the current study was small, further evaluation especially for chronic gHAT cases are necessary to evaluate the usefulness of blood-lysis method in the field setting.

In the present study, we established pan-*T*. *brucei* detecting CZC-LAMP HAT and rHAT-specific CZC-LAMP rHAT detecting the SRA gene in *T*. *b*. *rhodesiense*. Although the sensitivity was lower than that of CZC-LAMP HAT that targeted multi-copy number locus, the CZC-LAMP rHAT will be particularly useful when the parasite-reservoir animals and tsetse-flies are tested for the presence of human-infective *T*. *b*. *rhodesiense* in the rHAT endemic areas. Animals and tsetse-flies are often co-infected with non-human infective *Trypanosoma brucei brucei* in endemic areas, and differentiation of these subspecies is only possible using the SRA gene detecting molecular methods. Currently, the application of nucleic acid-based tests in reservoir animals and tsetse flies is limited to research purposes. Implementation of SRA detection method such as our CZC-LAMP rHAT in the veterinary and tsetse control section will considerably benefit the establishment of control strategies, that will result in improved human public health. Optimization and evaluation of CZC-LAMPs using animals and tsetse fly samples is necessary and currently ongoing.

In summary, we developed a novel procedure to make the dried-format LAMP test kit for the detection of HAT. The bio-inkjet printer enabled accurate printing of the LAMP reagents onto a test tube lid, and the stability and feasibility were demonstrated by analytical sensitivity assays and by evaluating the HAT patient samples. The developed LAMP test can offer sensitive and reliable diagnosis for HAT, which allows rapid point-of-care testing (POCT) and early treatment. The stability of the reagent at room temperature, the easy handing procedure, and the robustness are also beneficial for use in resource-poor settings. The developed semi-automated LAMP kit production procedure can also be applied to other LAMP tests to detect other infectious diseases as a POCT strategy.

## Supporting information

S1 Fig Inter-batch variability of CZC-LAMPs. Three independent batches (batches #D, #E #F) of CZC-LAMPs were assessed for their reaction speed (time of positivity) and sensitivity. Time of positivity determined by real-time PCR are plotted as blue dots. The dashed line presents the threshold of the reaction time to calculate sensitivity. a Indicated equivalent numbers of Trypanosoma brucei rhodesiense lysate per reaction (eq.) were used as templates. bThe positivity was judged at the 60-min (A; CZC-LAMP HAT) or 30-min (B; CZC-LAMP rHAT) incubation time threshold.
(TIF)
