# Supplementary figures and images for: Development of a bio-inkjet printed LAMP test kit for detecting human African trypanosomiasis
Source: PLoS Negl Trop Dis. 2020 Oct 22;14(10):e0008753. doi: 10.1371/journal.pntd.0008753 (PMC7608988; doi:10.1371/journal.pntd.0008753)

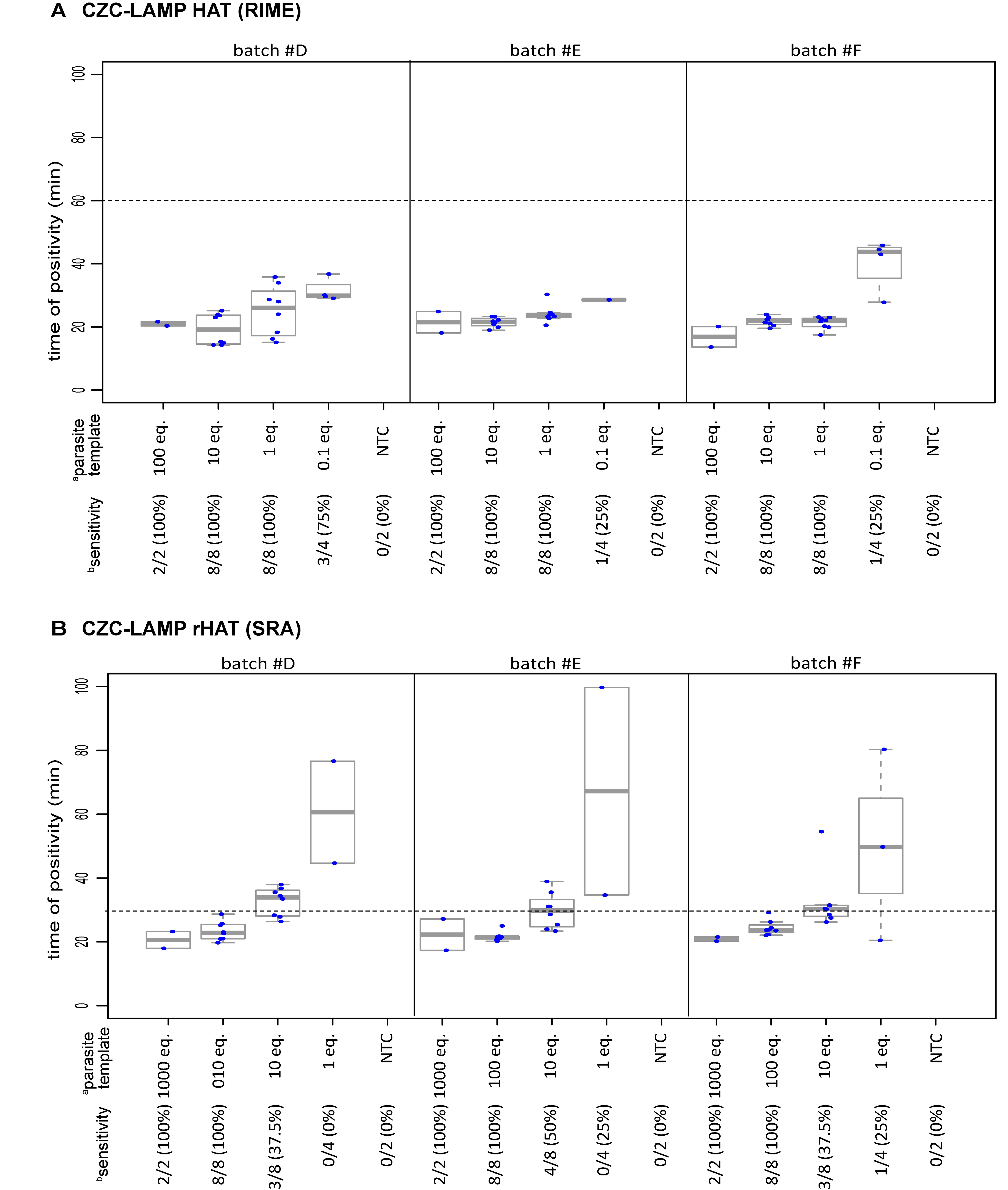

Supplement: S1 Fig — Time of positivity determined by real-time PCR are plotted as blue dots. The dashed line presents the threshold of the reaction time to calculate sensitivity. a Indicated equivalent numbers of Trypanosoma brucei rhodesiense lysate per reaction (eq.) were used as templates. bThe positivity was judged at the 60-min (A; CZC-LAMP HAT) or 30-min (B; CZC-LAMP rHAT) incubation time threshold. (TIF) [file pntd.0008753.s001.tif]
